# Dynamics of emergence and genetic diversity of dengue virus in Reunion Island from 2012 to 2022

**DOI:** 10.1371/journal.pntd.0012184

**Published:** 2024-05-20

**Authors:** Etienne Frumence, David A. Wilkinson, Raphaelle Klitting, Muriel Vincent, Nicolas Mnemosyme, Gilda Grard, Nicolas Traversier, Ghislaine Li-Pat-Yuen, Diana Heaugwane, Laurent Souply, Claude Giry, Marie-Claire Paty, Louis Collet, Local Laboratory Network, Patrick Gérardin, Fabian Thouillot, Xavier De Lamballerie, Marie-Christine Jaffar-Bandjee

**Affiliations:** 1 Centre National de Référence Arbovirus Associé, CHU de la Réunion Site Nord, Saint-Denis, Réunion, France; 2 Laboratoire de microbiologie, CHU de la Réunion-Site Nord, Saint-Denis, Réunion, France; 3 UMR ASTRE, CIRAD, INRAE, Université de Montpellier, Plateforme technologique CYROI, Sainte-Clotilde, Réunion, France; 4 Unité des Virus Émergents (UVE), Aix-Marseille Univ, IRD 190, INSERM 1207, Marseille, France; 5 CNR des Arbovirus, Marseille, France; 6 Santé Publique France, Saint Denis, Réunion, France; 7 Santé Publique France, Saint Maurice, France; 8 Centre Hospitalier de Mayotte, Mayotte, France; 9 INSERM CIC 1410, CHU de la Réunion, Saint-Pierre, Réunion, France; Faculty of Science, Ain Shams University (ASU), EGYPT

## Abstract

**Background:**

Dengue is a major public health concern in Reunion Island, marked by recurrent epidemics, including successive outbreaks of dengue virus serotypes 1 and 2 (DENV1 and DENV2) with over 70,000 cases confirmed since 2017.

**Methodology/Principal findings:**

In this study, we used Oxford Nanopore NGS technology for sequencing virologically-confirmed samples and clinical isolates collected between 2012 and 2022 to investigate the molecular epidemiology and evolution of DENV in Reunion Island. Here, we generated and analyzed a total of 499 DENV1, 360 DENV2, and 18 DENV3 sequences. By phylogenetic analysis, we show that different genotypes and variants of DENV have circulated in the past decade that likely originated from Seychelles, Mayotte and Southeast Asia and highly affected areas in Asia and Africa.

**Conclusions/Significance:**

DENV sequences from Reunion Island exhibit a high genetic diversity which suggests regular introductions of new viral lineages from various Indian Ocean islands. The insights from our phylogenetic analysis may inform local health authorities about the endemicity of DENV variants circulating in Reunion Island and may improve dengue management and surveillance. This work emphasizes the importance of strong local coordination and collaboration to inform public health stakeholders in Reunion Island, neighboring areas, and mainland France.

## Author summary

This study, conducted from 2012 to 2022, aimed at understanding the dynamics and genetic diversity of dengue virus on Reunion Island, a French overseas department located in the southwestern Indian Ocean, which is regularly affected by dengue epidemics. Several thousand clinical isolates and patient samples were collected from across the island and sequenced, resulting in 877 new genetic sequences of dengue virus. Our results show significant genetic diversity among dengue virus variants that circulated on the island over the past decade. This diversity indicates the simultaneous presence of multiple lineages and closely related viruses, which may have resulted from regular introductions of new variants. Our phylogenetic analyses show that the sequences detected in Reunion Island may have originated from other Indian Ocean islands, such as Seychelles and Mayotte, as well as from Asian regions where dengue virus circulates.

## 1. Introduction

Dengue is the most prevalent arthropod-borne viral infection, annually affecting between 100–400 million individuals worldwide, with roughly half of the world’s population at risk of contracting the disease [1,2]. Dengue is caused by dengue virus (DENV), which, like other mosquito-borne viruses of medical relevance including Zika, Japanese Encephalitis, Yellow Fever, or West Nile viruses, belongs to the *Flavivirus* genus. DENV is primarily transmitted to humans through the bites of infected *Aedes* mosquitoes, particularly *Aedes aegypti* and *Aedes albopictus*.

DENV infection causes a polymorphous disease that ranges from mild flu-like symptoms known as dengue fever, to severe forms, formerly designated as dengue hemorrhagic fever and dengue shock syndrome, now designated under the definition of severe dengue (severe plasma leakage, severe hemorrhages and/or severe organ involvement), which can be fatal if not promptly and effectively treated [3–5]. The disease burden of dengue is difficult to estimate because up to 75% of cases are inapparent, either asymptomatic or oligosymptomatic [1,6].

There are four distinct serotypes of DENV, known as DENV1, DENV2, DENV3, and DENV4. Immunity against one serotype does not provide a durable protection against the others, and in some cases, a secondary infection with a different serotype can result in more severe symptoms that may be linked to antibody-dependent enhancement [3,7]. Currently, there is no specific treatment for dengue available. The Dengvaxia and Qdenga vaccines (the latter having been licensed by the European Commission since December 8, 2022 for use in people over the age of 4) are not used in France [8]. Recommendations for use will be issued by the French Health Authority in spring 2024 [9]. At this stage, prevention is deemed to be the most effective strategy for controlling the virus, including measures such as protection against mosquito bites and the eradication of mosquito populations [10].

DENV is a single-stranded positive-sense RNA virus, that has a genetic organization characterized by a single open reading frame of approximately 11kb, encoding for three structural proteins (C, prM, and E) and seven non-structural proteins (NS1, NS2A, NS2B, NS3, NS4A, NS4B, and NS5) [11]. RNA viruses such as DENV have a high rate of mutation during genome replication, leading to high genetic variability [12,13]. The four serotypes of DENV are 30–35% divergent at the amino acid level when compared to each other. Within each serotype, several genotypes have been described, typically differing by approximately 3% at the amino acid level and not exceeding a 6% difference at the nucleotide level [12].

In the Indian Ocean region, dengue is a tremendous public health concern, where it is thought to have gone towards endemicity [14]. Reunion Island, a French overseas department situated at the crossroads of South-East Africa, Madagascar, the Comoros, and Asia in the South-Western Indian Ocean (SWIO) region, has a long history of dengue epidemics dated from the 70s, with a high-magnitude DENV2 outbreak in 1977–78 that affected approximately 30% of the population [14]. Subsequently, the island experienced 40 years of low-level transmission with microepidemics and sporadic seasonal autochthonous cases until 2018–2019, when it faced new epidemics and perennial transmission sustained first by DENV2, and then by DENV1 in 2020–2021 [15–20]. The control of dengue in Reunion Island relies on vector control measures, such as the use of insecticides and the destruction of mosquito breeding sites. As part of the effort to develop effective alternatives to existing vector control strategies, the sterile insect technique (SIT) is currently being tested on Reunion, following a multi-year feasibility program established by the French Research and Development Institute (IRD) in collaboration with national and international partners [21].

To address the growing threat of dengue in Reunion Island, the associated National Reference Center (NRC) for Arboviruses at Reunion Island Hospital has been actively conducting molecular surveillance for DENV for over a decade. In this study, virologically-confirmed samples (DENV positive RT-PCRs) and clinical isolates collected between 2012 and 2022 were sequenced using the Oxford Nanopore next-generation sequencing (NGS) technology to investigate the molecular epidemiology and evolution of DENV on Reunion Island. The valuable insights gained from this work may inform and guide public health interventions aimed at effectively controlling the spread of the disease, ultimately contributing to better management and surveillance of dengue.

## 2. Materials and methods

### Ethics statement

This study was part of national public health surveillance program of the National Reference Center (NRC) for Arboviruses supervised by the National Public Health Agency (Santé Publique France, SPF). Therefore, as an epidemiological record, consultation with ethics committee was not required. Samples involved in this study were chosen among human plasma and serum specimens received as part of routine standard diagnostic and expertise activities of the associated arboviruses NRC in Reunion Island’s Hospital. The need for informed consent was waived because of the retrospective and anonymous nature of the study. No additional clinical specimens were collected for the purpose of the study. Human samples and cultures originating from human samples were anonymized, with no or minimal risk to patients within the terms of the European General Data Protection Regulation and the French National Commission on Informatics and Liberty (CNIL).

### Sample collection

The arbovirus surveillance system in Reunion Island has been already described elsewhere [16,20]. Briefly, as a mandatory notifiable disease under surveillance, it is recommended to test all individuals presenting dengue-like symptoms. Laboratory confirmation of DENV suspected cases is performed through the detection of viral genome by RT-PCR, which is considered the gold standard for samples collected within 5 days post symptom onset. This procedure is carried out by all medical biology laboratories across Reunion Island, including private city laboratories and public hospital lab centers. Subsequently, almost all cases reported during interepidemic phases and a geographically representative set of samples reported during epidemic phases, as well as all possible imported cases, severe and/or atypical cases, and fatal cases are sent to the microbiology laboratory of the Reunion Island Hospital for serotyping and further analysis since it became an associated arboviruses NRC in 2012. Notably, samples from the neighboring island of Mayotte may also be sent to the associated Arboviruses NRC for analysis, as it also serves as a regional reference laboratory.

During non-epidemic periods, samples with sufficient viral load were systematically isolated on Vero cells after one or two 5–7 day passages in a BSL3 facility. However, during epidemic periods, the large number of samples received prevented systematic isolation. Clinical specimens and isolates were stored at -80°C. Sequence data were preferentially generated from viral culture supernatant for all samples where viral isolation was successful. During non-epidemic phases, all positive samples or isolates were systematically selected for sequencing based on a Ct value of <30 to maximize genome coverage. During the epidemic periods from 2018 to 2021, sample selection was pseudo-random, with temporal preferential sampling favoring pre-epidemic periods and based on a Ct value of <30.

### Serotyping of dengue virus

Viral RNA was extracted from sera or plasma using the NucliSENS easyMAG kit (Biomerieux) according to the manufacturer’s recommendations. DENV serotypes were determined using an in-house one-step quantitative RT-PCR on Roche LightCycler 480 Real-Time PCR System (Roche, Germany) according to a previously published protocol [22].

### Sequencing analysis

Genome sequencing of DENV was performed directly on viral RNA of positive cases using the amplicon-tiling protocol for Oxford Nanopore Technologies (ONT) sequencing platform [23,24]. Viral RNA was extracted from 200 μl of clinical isolates collected between 2012 and 2018, as well as from serum and plasma samples from patients between 2019 and 2022, using the NucliSENS easyMAG kit on easyMAG or eMAG automation systems (Biomerieux), using the generic extraction protocol, according to the manufacturer’s instruction. RNA was used as a template for cDNA synthesis using Lunascript RT supermix kit (New England Biolab, MA, USA). Nuclease-free water was used as a blank control sample.

Serotype-specific multiplex PCRs were then performed in two pools per sample, using primer schemes designed to amplify overlapping fragments of ~1200bp. Primer schemes were developed using the Primal Scheme software package [23], and are detailed in S1 Table. PCR reactions were performed using the Q5 HS high fidelity 2X master mix (New England Biolabs, MA, USA). Pooled amplicons were then quantified using the 1x dsDNA Broad range Quant-it kit (ThermoFisher) and normalized to 150 ng DNA per well using a *Zephyr* G3 *NGS* benchtop liquid handler (PerkinElmer). DNA library preparation was performed using the Rapid Barcoding Kit 96 (SQK-RBK110.96) according to the manufacturer’s recommendations and loaded on R9.4.1 flow cells (FLO-MIN106D) on the Oxford Nanopore GridION platform. Raw data was base-called using the latest super-accurate configuration, filtered for high-quality reads (mean Q-score > 10) and demultiplexed using the MinKNOW software package. Bioinformatic generation of consensus sequences was conducted using the ARTIC network’s field bioinformatics pipeline (https://github.com/artic-network/fieldbioinformatics) using DENV1 (NC_001477.1), DENV2 (NC_001474.2) and DENV3 (NC_001475.2) reference genomes.

Our amplicon-based sequencing technique allows for the coverage of more than 97% of the viral genomes, except for the ends not covered by our primers, resulting in newly generated genomes that do not contain the complete 5’/3’-UTR regions. Nearly complete genomes were aligned in Geneious Prime software version 2023.1.2 with MAFFT multiple aligner version 1.5.0, using default parameters, trimmed from free-end gaps, manually curated and annotated. Genomes with >30% missing data or a median read depth lower than 300× were excluded from further analyses.

### Genotype assignment and phylogenetic analyses

To initially define the genotype of the newly assemble DENV genomes, the Dengue Virus Typing tool from Genome detective was used (https://www.genomedetective.com/app/typingtool/dengue/ accessed on February 2023) [25]. To refine the genotype assignment, we conducted phylogenetic analysis using MAFFT version 7.310 and the maximum-likelihood IQTree version 1.6.12 with 1,000 ultrafast bootstrap replicates on all available DENV Envelope (E) gene nucleotide sequences from the NCBI GenBank database (as of September 2023). To investigate the origins and spatial dynamics of DENV in Reunion Island, we generated maximum-likelihood trees by analyzing the near-complete genomes obtained in this study along with all available DENV1, DENV2, and DENV3 sequences from the GenBank database with lengths exceeding 5,000 nucleotides (as of September 2023). These datasets, which included approximately 5,000 DENV1 sequences, 4,200 DENV2 sequences, and 2,100 DENV3 sequences, were cleaned of sequences containing excessive ambiguities, extremely divergent sequences, or those that had been misassigned. We aligned the sequences using MAFFT and estimated Maximum-Likelihood (ML) phylogenies using IQTree with the best-fitting model and 1,000 ultrafast bootstrap replicates. Trees and extracted subtrees focusing on specific sequences were annotated and visualized using Figtree v1.4.4 and iTOL v6.8.1 (http://itol.embl.de).

## 3. Results

### 3.1. Epidemiological context

From 2012 to 2022, the arboviruses associated NRC successfully serotyped over 4,700 positive DENV cases by RT-PCR, originating from all the laboratories in Reunion Island. These samples represented 6.4% of the over 73,000 DENV confirmed cases during this period. Among these cases, more than 1,100 samples were sequenced using an amplicon-based NGS nanopore sequencing approach to achieve a more precise identification of the circulating variants. Out of these, 877 had a coverage greater than 80% and a median read depth greater than 300x and were included in this study. Overall, our implementation of a cost-effective and time-efficient rapid sequencing protocol using Oxford Nanopore Technology and specific primer panels for each dengue serotype allowed us to sequence a total of 499 DENV1, 360 DENV2, and 18 DENV3 samples from the past 10 years. The sequences had a mean coverage of 96.2% and a median read depth of 1500x ±591, allowing us to generate nearly complete DENV genomes. To better describe the molecular epidemiology of dengue in Reunion Island, the results were segregated into three periods: a low viral circulation period between 2012 and 2016, a high circulation period of DENV2 from 2017 to 2020 that overlapped with a third period from 2019 to 2022, featuring the circulation of DENV1 and, to a lesser extent, DENV3.

### 3.2. Genetic diversity of DENV in Reunion Island from 2012–2016

**Fig 1:**
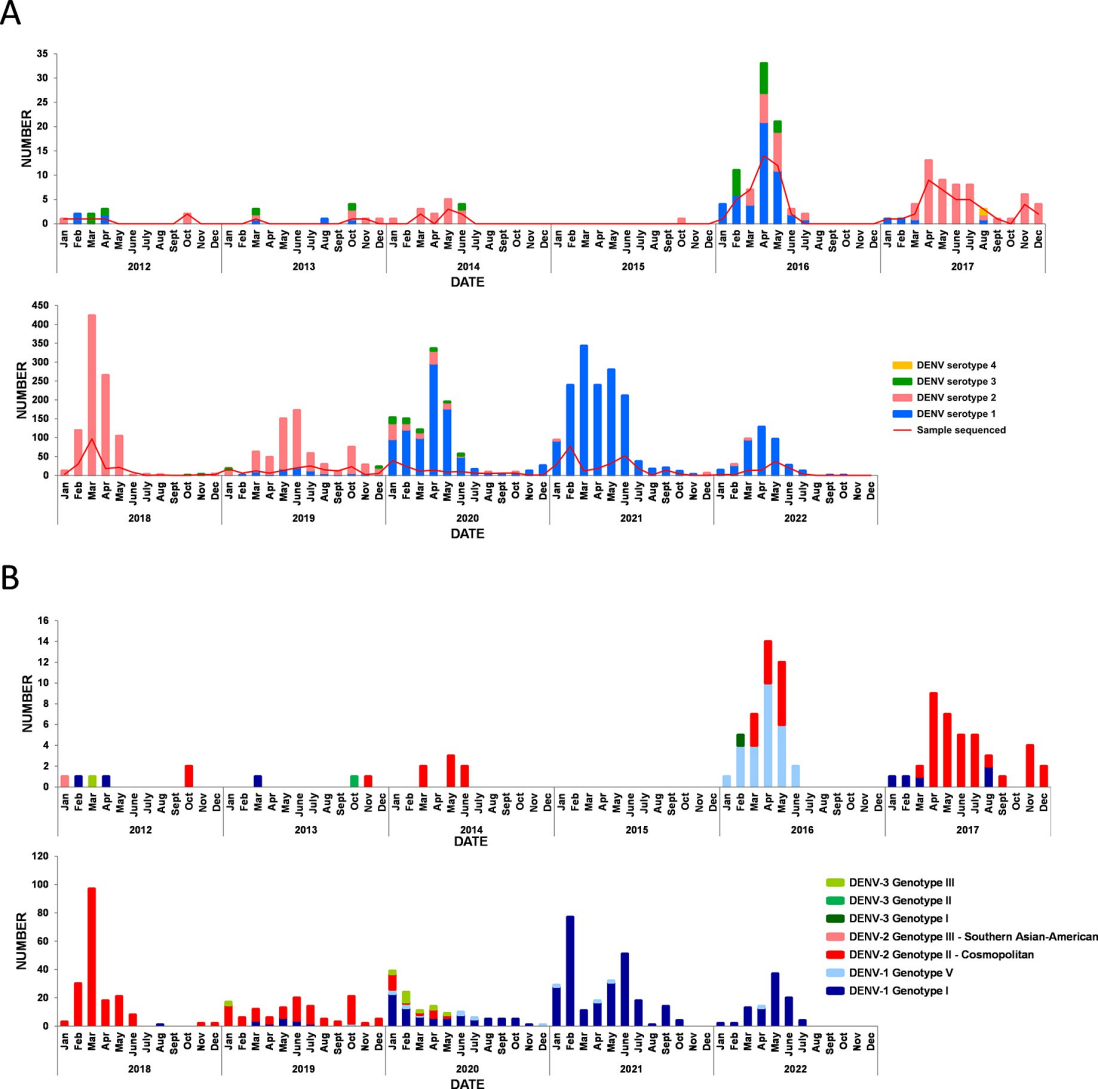
Summary of the serotyping and genotyping of dengue virus variants detected in Reunion Island from 2012 to 2022. Panel A shows the distribution of serotypes as determined by RT-PCR (n = 4,792), along with the number of sequenced samples in this study. Panel B shows the genotypes of DENV sequences analyzed in this study (n = 877).

**Fig 2:**
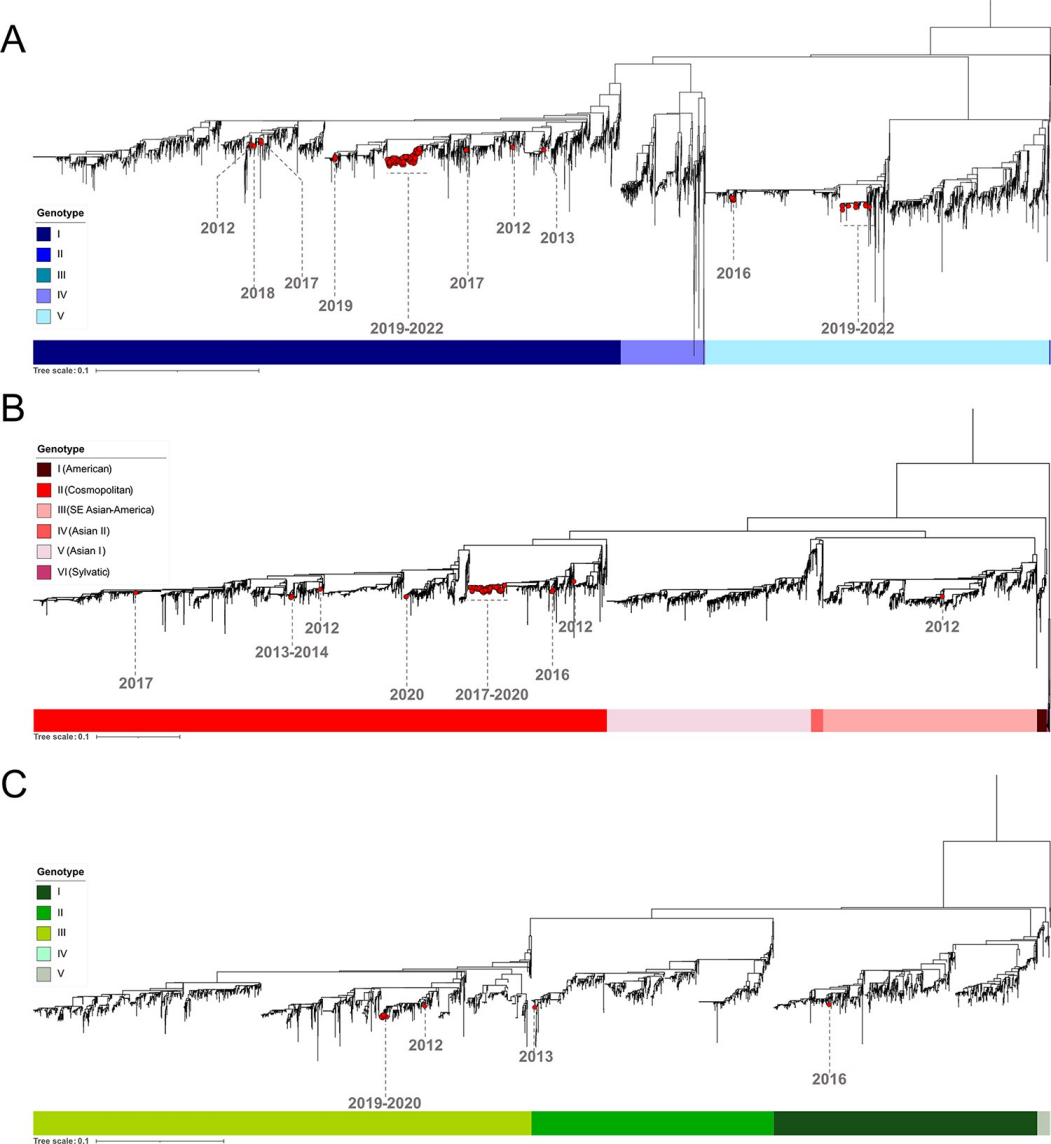
Genotyping of DENV1 (A), DENV2 (B), and DENV3 (C) sequences from Reunion Island from 2012 to 2022 based on E gene sequences. Maximum likelihood (ML) trees were constructed to investigate the phylogenetic relationships among these sequences using all E sequences available in the NCBI GenBank database. The sequences from Reunion Island obtained in this study are represented by red dots.

The circulation of dengue virus on the island was notably low during the period of 2012 to 2016 (Fig 1). DENV serotypes 1, 2 and 3 were identified during this time, with a higher percentage of imported cases based on the data provided by the French Public Health Agency (SPF) [14]. Among the samples with sufficient viral load, the majority were successfully isolated and sequenced. A total of 57 almost complete genome sequences were obtained, as shown in Fig 1, which corresponds to more than 16% of all the virologically confirmed cases on the island during that period. Our results for the period from 2012 to 2014 indicate the presence of multiple genotypes of DENV that were either imported or circulated on the island, including three variants of genotype I of DENV1, one genotype III Southern Asian-American variant and ten genotype II cosmopolitan variants of DENV2, as well as one genotype III and one genotype II variants of DENV3 (Figs 1B and 2).

**Fig 3:**
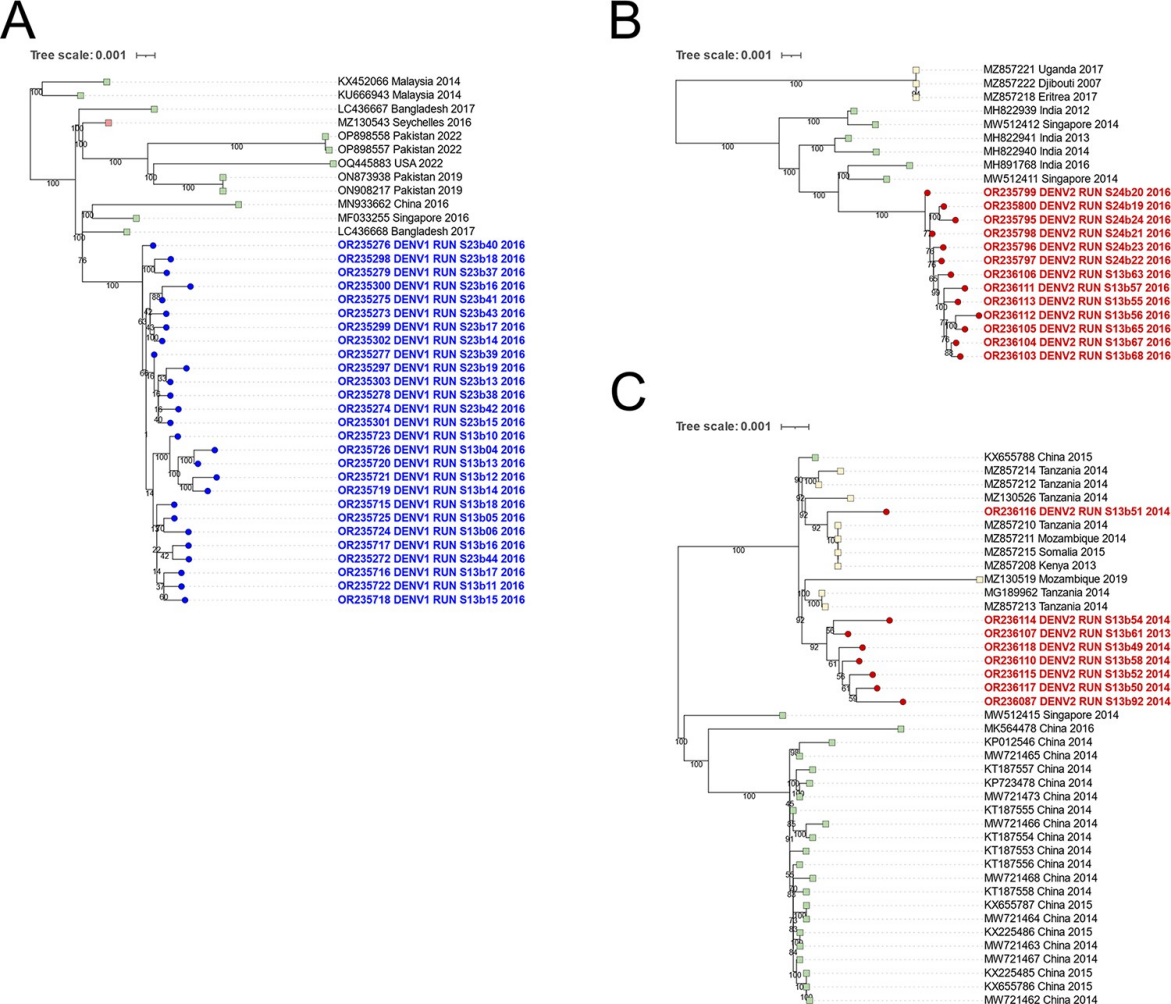
Phylogenetic relationships of DENV1 (A) and DENV2 (B and C) from 2013–2016 in Reunion Island. These subtrees, taken from S1 Fig, were generated by constructing ML trees using all DENV1 or DENV2 sequences from the GenBank database with lengths exceeding 5,000 nucleotides. They highlight the Reunion Island sequences from this study with circles: the 2016 DENV1 sequences in blue (A), the 2016 DENV2 sequences in red (B), and the 2013–2014 DENV2 sequences in red (C). The closest sequences from the GenBank database are represented by squares.

The number of autochthonous cases increased significantly in 2016, with the circulation of DENV1 genotype V and DENV2 genotype II cosmopolitan (Fig 1B). Additionally, twelve DENV3 cases were detected in 2016 by serotyping, with only one sample successfully genotyped, belonging to genotype I. Maximum likelihood trees were constructed to investigate the phylogenetic relationships among these sequences and all DENV sequences with lengths exceeding 5,000 nucleotides available in the NCBI GenBank database (S1 Fig). As shown in Fig 3A, DENV1 genotype V sequences from 2016 were found to be most similar to Asian sequences from Bangladesh and Singapore (up to 99.73% identity), as well as sequences originating from Seychelles from the same period (reaching up to 99.71% identity).

Regarding the DENV2 sequences from 2016, they belonged to genotype II Cosmopolitan and exhibited the highest similarity (up to 99.46% identity) to sequences from Singapore and India (Fig 3B). Notably, the DENV2 sequences circulating in 2016 were distinct from those observed in 2013–14 (Fig 2B), which included a sample imported from Tanzania and showed a closer relationship to African sequences from Tanzania with up to 99,82% sequence identity (Fig 3C).

### 3.3. The DENV2 epidemic: 2017–2020

**Fig 4:**
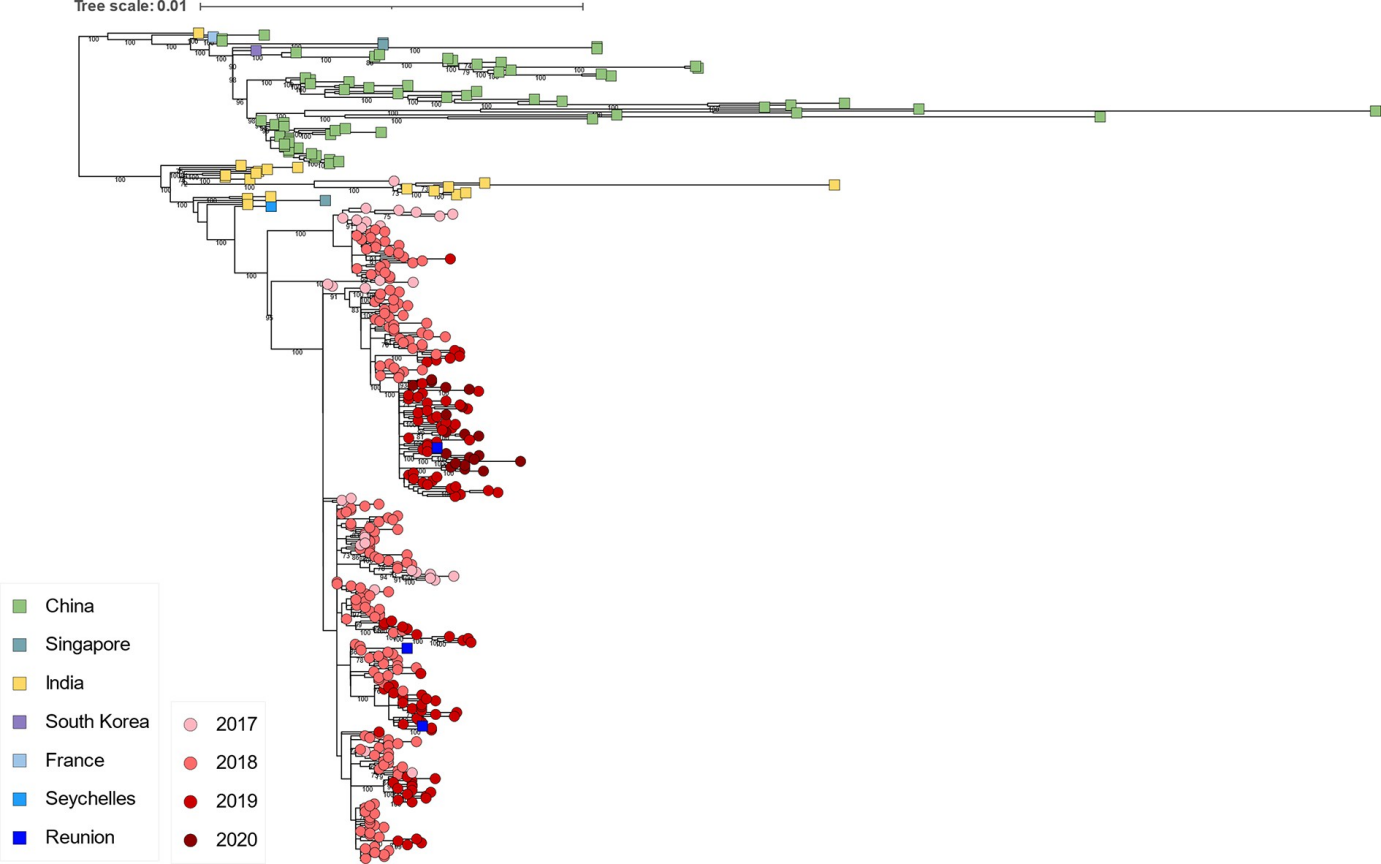
Phylogenetic relationships of DENV2 sequences from 2017 to 2020. The subtree, taken from S1B Fig, was created by constructing a ML tree using all DENV2 sequences from the GenBank database that were longer than 5,000 nucleotides. It highlights the 2017–2020 DENV2 sequences from this study, which are depicted with circles and a red gradient according to their collection dates. The closest sequences from the GenBank database are presented with squares and color-coded according to their countries of origin.

A major epidemic of DENV2 occurred on Reunion Island between 2018 and 2019, with a total of 6,770 confirmed cases reported in 2018 and a subsequent increase to 18,217 cases in 2019 [14,16]. To characterize this epidemic with precision, we randomly sequenced and analyzed 336 samples of DENV2 during this period, including DENV2 sequences from 2017. Genotyping analysis revealed that the 2017–2020 epidemic was caused by a single DENV2 genotype, the genotype II cosmopolitan lineage (Figs 1B and 2B). Based on phylogenetic inference using sequences from Reunion combined with a set of publicly available DENV2 sequences, we observed that the lineage of DENV2 that circulated between 2017 and 2020 exhibited a high degree of similarity and did not group with the sequences of previous DENV2 variants that circulated in 2014 and 2016 on Reunion Island (Fig 2B). As shown in Fig 4, several clusters of closely related cosmopolitan lineages circulated on the island in 2017, suggesting multiple independent introduction events. These sequences were found to be most closely related to variants from India, Singapore, and Seychelles (up to 99.73% identity). Of note, three sequences in 2017 were from imported cases: one from Seychelles and one from Sri Lanka in April, and one from India in November. Additionally, public health authorities reported six other importations that year from various countries, including Indonesia, Myanmar, and Thailand [11]. Notably, during this epidemic, we detected the introduction of two distinct variants in 2020 that exhibited the highest similarity (99.81% identity) to sequences from French Polynesia and New Caledonia (Fig 2B).

### 3.4. Genetic characteristics of the DENV Epidemic from 2019 to 2022

In 2019, co-circulation of DENV1 was detected alongside the ongoing DENV2 epidemic. Starting from 2020, a complete replacement of DENV2 by DENV1 occurred, leading to a new epidemic that persisted until the end of austral summer 2021 (Fig 1). During this period, we sequenced 463 randomly selected DENV1 samples, which represented approximately 1% of the reported cases on the island. Our results show that two genotypes of DENV1, genotype I and genotype V, co-circulated from 2019 to 2022 (Figs 1B and 2A). The genotype I was largely predominant, but genotype V, detected since 2019, persisted at low levels (around 3% of the sequenced DENV1) until 2022. In addition, low-level circulation of DENV3 was also detected during this period, predominantly associated with the genotype III.

### 3.5. The DENV1 genotype I Epidemic: 2019–2022

**Fig 5:**
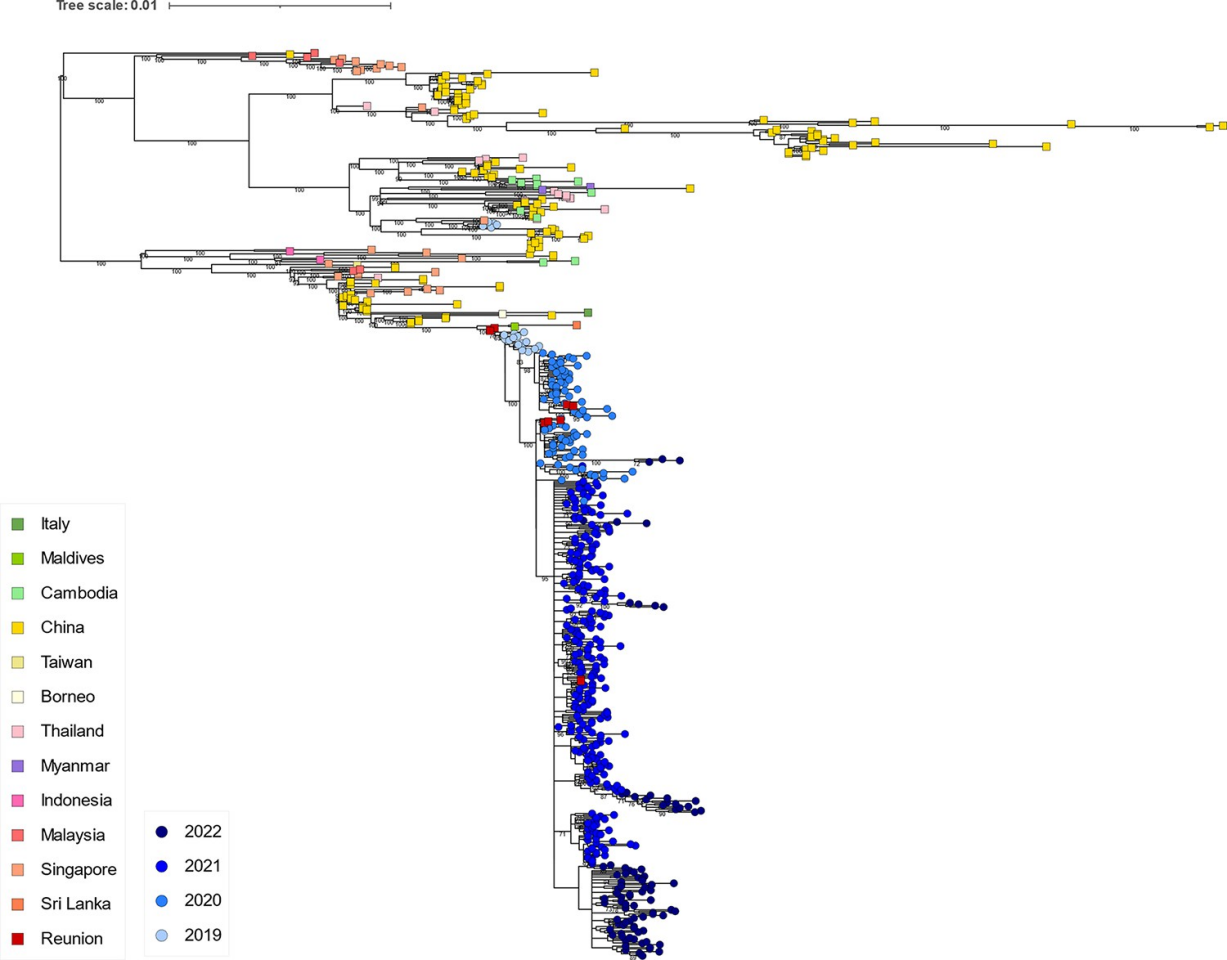
Phylogenetic relationships of DENV1 genotype I sequences from 2019 to 2022. The subtree, taken from S1A Fig, was generated by constructing a ML tree using all DENV1 sequences from the GenBank database that were longer than 5,000 nucleotides. It highlights the 2019–2022 genotype I DENV1 sequences from this study, which are depicted with circles and a blue gradient corresponding to their collection dates. The closest sequences from the GenBank database are presented with squares and color-coded according to their countries of origin.

Phylogenetic analysis revealed that at least two clusters of DENV1 genotype I sequences circulated on Reunion Island in 2019, which suggested multiple introduction events (Fig 5). The cluster of five variants closely related to sequences from Southeast Asia (with up to 99.80% identity) appears to have not circulated in the following years. The variants responsible for the DENV1 epidemic from 2020 to 2022 exhibited similarities to sequences from China, Sri Lanka, and the Maldives (with up to 99.88% identity). It is noteworthy that these sequences did not cluster with sequences from the DENV1 genotype I variants previously detected on Reunion Island in 2012–2013 and 2017–2018 (Fig 2A).

### 3.6. Genetic comparison of DENV1 genotype V from Reunion and Mayotte Islands

**Fig 6:**
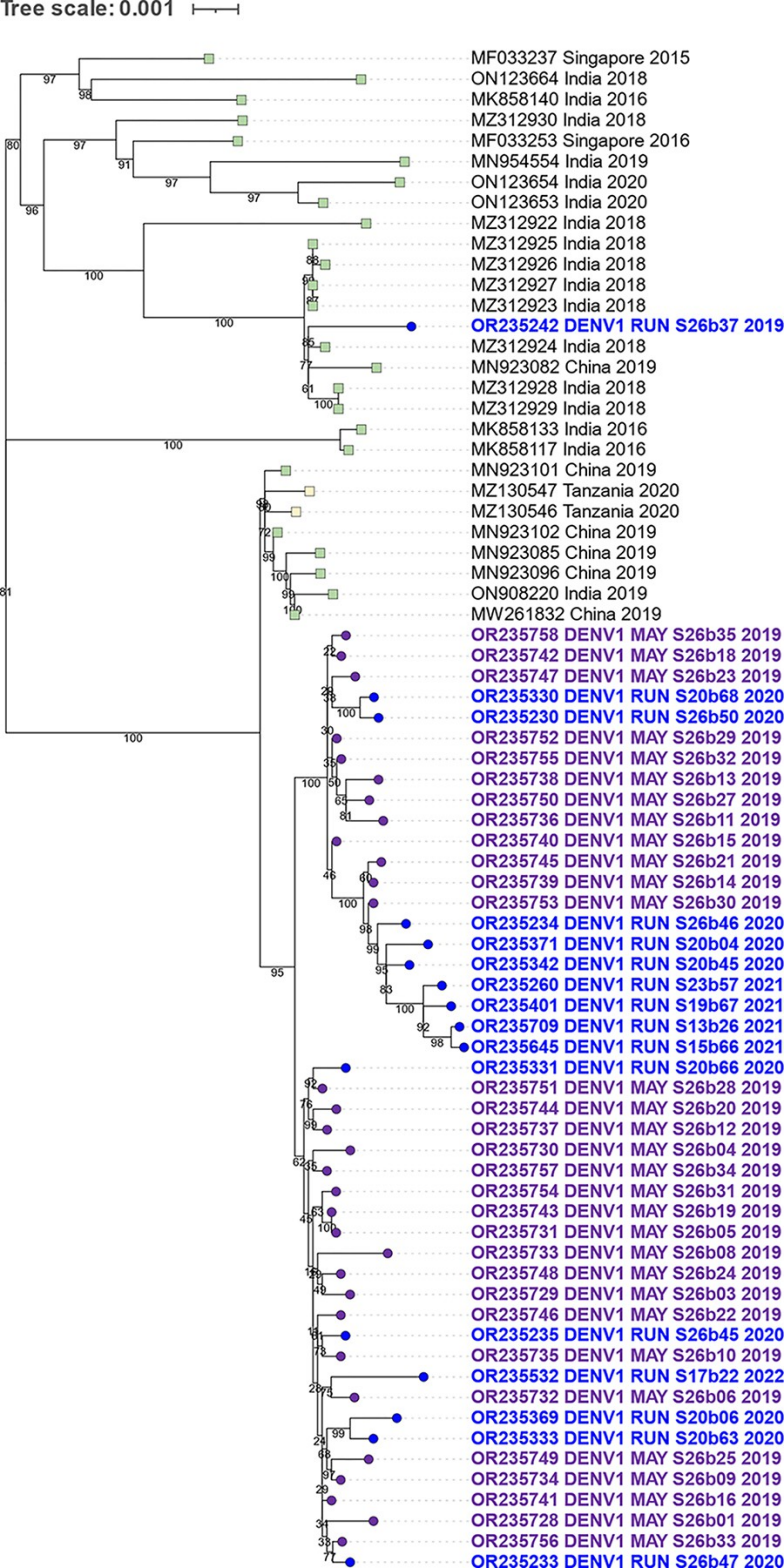
Genetic relationships of DENV1 genotype V sequences from 2019 to 2022. The subtree, taken from S1A Fig, was generated by constructing a ML tree using all DENV1 sequences from the GenBank database that were longer than 5,000 nucleotides. It highlights the 2019–2022 genotype V DENV1 sequences from Reunion Island in blue rounds and from Mayotte Island in purple rounds. The closest sequences from the GenBank database are represented by squares.

Regarding the genotype V sequences of DENV1 detected at a low level from 2020 to 2022, our phylogenetic analysis reveals that these sequences shared the highest similarity (up to 99.80% identity) with sequences from China, India and Tanzania in 2019–2020 (Fig 6). These sequences were distinct from previously identified genotype V sequences from Reunion Island in 2016 (Fig 2A). Notably, only one sequence from 2019 (accession OR235242, variant DENV1_RUN_S26b37_2019) was relatively distant from the others. Within these samples of DENV1 genotype V, four sequences were imported from the islands of Mayotte and Comoros through recent travels. Given the number of imported cases, we decided to analyze samples directly sourced from Mayotte. In 2019, Mayotte hospital detected multiple positive cases of DENV, which led to the retrospective submission of 31 samples to the associated NRC for arboviruses, where they underwent serotyping and further characterization. We found that all samples from Mayotte belonged to the genotype V of DENV1. Phylogenetic analysis confirmed that the sequences from Mayotte exhibited a high degree of similarity, with identities that could exceed 99.9%, to those from Reunion Island (Fig 6). Taken together, these findings suggest that the genotype V variant, which circulated sporadically and/or was regularly imported to Reunion Island from 2020 to 2022, may have originated directly from Mayotte Island, where this lineage was circulating in 2019.

### 3.7. Analysis of DENV3 that circulated on Reunion in 2019 to 2020

**Fig 7:**
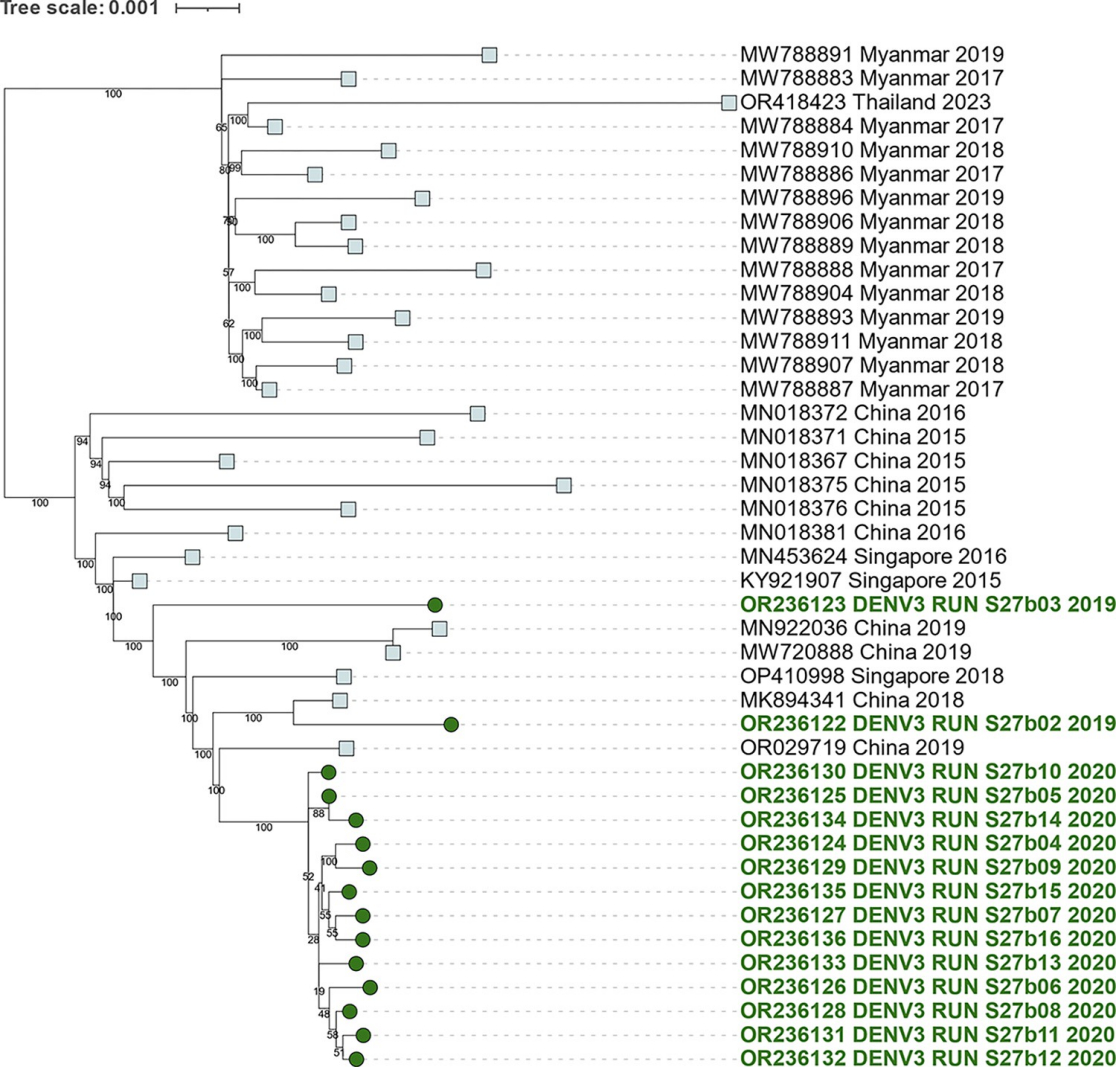
Genetic relationships of DENV3 genotype III sequences from 2019 to 2020. The subtree, taken from S1C Fig, was generated by constructing a ML tree using all DENV3 sequences from the GenBank database that were longer than 5,000 nucleotides. It highlights the 2019–2020 DENV3 sequences from this study, represented in green rounds. The closest sequences from the GenBank database are represented by squares.

Between 2019 and 2020, there was a notably restricted temporal and spatial circulation of DENV3 reported (Fig 1). Phylogenetic analyses revealed that these sequences belonged to genotype III of DENV3. These sequences were quite distinct from the genotype III sequence obtained in 2012 in Reunion Island (Fig 2C). Instead, they exhibited similarities (up to 99,70% identity) with sequences of Asian origin particularly those from China from the same period (Fig 7).

## 4. Discussion

In response to the recent significant increase in dengue-case incidence on Reunion Island [14,16,17,20], we have retrospectively and systematically applied Oxford Nanopore-based genomic sequencing protocols to viruses identified over a ten-year period as part of routine medical diagnostics. Through these analyses, we aimed to gain insights into the origins, potential introductions, and endemicity of dengue viruses on the island, given its unique geographical position at the crossroads of East Africa, Madagascar, the Comoros, and Asia. In this study, we provide compelling evidence of the remarkable genetic diversity among DENV genotypes circulating in Reunion Island over the past decade. Our findings reveal the presence of multiple DENV genotypes, each exhibiting substantial diversity in terms of variants and lineages.

Our serotyping results reveal the co-circulation of up to three different serotypes of DENV on Reunion Island during the same year, as observed in 2016, 2019, and 2020 (Fig 1A). Within these serotypes, we detected at least two genotypes of DENV1 (genotype I and V) and two genotypes of DENV2, with genotype II Cosmopolitan being the most prevalent. Among DENV3 variants, we identified at least three different genotypes (genotype I, II, and III), although its circulation remained very limited. During the study period, we found only one case of DENV4, but sequencing was not possible due to the low viral load. Furthermore, our results demonstrate the co-circulation of two distinct genotypes during the same epidemic period. Specifically, from 2020 to 2022 we observed low-level circulation of DENV1 genotype V, while genotype I was dominant and widely distributed. These findings highlight the importance of representative sample sizes to capture the full range of the genetic diversity and identify emerging or less prevalent lineages. During epidemic periods, we have observed the introduction and simultaneous circulation of closely related viral clusters. Some of these clusters may have persisted for months and continued in the following years, while others did not, as observed with DENV2 between 2017 and 2020, and with DENV1 in 2019. These observations suggest multiple introductions of closely related viral lineages, with variations in their ability to establish sustained transmission within the population. Exploring the factors influencing the persistence or disappearance of these clusters could provide valuable insights into the dynamics of DENV transmission and guide targeted control strategies.

Our findings also demonstrate the importance of continuous monitoring of viral genetic sequences during epidemics, as these viruses undergo continuous evolution and accumulate mutations over years of circulation. The DENV, being an RNA virus, is known to have a high mutation rate, as supported by our results [26–28]. Using TreeTime, we conducted a preliminary evaluation of substitution rates for epidemic DENV sequences from Reunion Island, resulting in an estimated rate of 6.36 x 10^−4^ substitutions/site/year for the 2017–2020 DENV2 sequences and 7.39 x 10^−4^ substitutions/site/year for the DENV1 sequences from 2019 to 2022. These mutations, which accumulate over time, contribute to the genetic diversity and evolutionary dynamics of the viruses, potentially leading to the emergence of new variants and influencing their virulence, transmission efficiency, and immune evasion capabilities [12,26–28].

Furthermore, our phylogenetic analyses using virus sequences from public databases provide valuable insights into the potential origins of virus importations to Reunion Island. Our observations indicate that several DENV variants that circulated in Reunion Island may have Asian origins, likely originated from hyperendemic areas of dengue such as India or China [1]. For example, the analysis of DENV1 sequences from 2019, DENV2 sequences from 2016 and 2017, and DENV3 sequences from 2019 suggested possible Asian origins. Additionally, we found evidence of a potential connection between of DENV1 variants from 2016 and DENV2 variants from 2017 with lineages from the Seychelles, an archipelago in the Mascarene region close to Reunion Island as already suggested by others [15]. Furthermore, the potential introduction of genotype V of DENV1 from Mayotte Island and/or the Comoros Islands indicates a possible inter-island spread of DENV genotypes within the Indian Ocean region. The close geographical proximity and frequent travel between these islands provide opportunities for the exchange and dissemination of DENV lineages as well as other arboviruses like the chikungunya virus, which circulated in Reunion Island and Mayotte in 2005–2006 [14,29,30]. Understanding these interconnections and monitoring the movement of viruses between these islands is crucial for effective surveillance and control strategies. By considering these cross-border dynamics, public health authorities could implement coordinated efforts to prevent and mitigate the spread of dengue in the region.

The potential introduction of DENV from other French overseas departments and territories where DENV has been circulating is also plausible, as observed in 2020 with samples of DENV2 that were potentially imported from the French South Pacific islands. Our findings also suggest the possible involvement of neighboring African countries in the introduction and circulation of DENV genotypes on Reunion Island, as shown by sequences of DENV2 dated from 2014 that may have been imported from Tanzania. It is however important to interpret these results with caution, as the sequence data available in public databases used in this study may not be comprehensive enough and with sequences lacking for many regions of interest with known virus circulation such as the Comoros Islands, Mauritius, or Madagascar.

In recent years, the circulation of DENV has extended beyond tropical regions, thanks to the combination of global climate change and the continued expansion of the tiger mosquito (*Aedes albopictus*) into temperate regions, including Europe where the disease was previously not endemic [31,32]. In 2022, a total of 378 imported dengue cases was reported in mainland France, with the identification of eleven transmission clusters and 66 autochthonous dengue cases [33,34]. Notably, the NRC for Arboviruses in Marseille detected at least four cases of DENV1 in the south of France that were linked to the DENV1 genotype I variant from the 2020–2022 epidemic on Reunion Island [33,34]. This suggests that Reunion Island could serve as a source for the introduction of new DENV variants into mainland France.

The COVID-19 pandemic has underscored the importance of global genomic surveillance and the necessity of equitable data-sharing systems and international collaboration in confronting pathogen threats. It is now imperative to set up regular and sustainable large-scale surveillance for high-impact arboviruses like DENV, with the assistance of advanced data-sharing platforms and high-quality curated databases, to facilitate the tracking of lineage prevalence and identification of viral clusters with the highest epidemic potential [35]. In this context, ONT devices have emerged as a promising tool for viral genomic surveillance, which offers several advantages such as simplicity, the ability to generate long-read sequences, and cost-effectiveness. This effectiveness may be particularly relevant and scalable for developing countries. It is however important to acknowledge that ONT devices might have lower read-level sequencing accuracy compared to short-read platforms like Illumina devices, with individual sequences having sequencing efficiency as low as one error every ten bases (Q10). Notwithstanding, consensus sequence quality, critical for phylogenomic analysis and conclusions, is not presumed to be adversely affected by this choice of sequencing technology, as data suggests that Nanopore-generated consensus sequences could be considered reliable above read depths of 60x [36].

In summary, while Reunion Island has faced tremendous challenges with major DENV outbreaks in recent years, our results demonstrate that DENV variants have not become endemic and exhibit high genetic diversity due to multiple introductions from neighboring islands and regions, including Asia and Africa. To effectively monitor and control the spread of these viruses, strong local coordination and collaborative monitoring efforts are essential. By sharing data and resources, implementing comprehensive surveillance systems, and fostering interregional cooperation, we should better understand the dynamics of DENV transmission and enhance control strategies. These efforts are critical to mitigate the impact of dengue outbreaks and protect public health in Reunion Island, other neighboring islands of the SWIO region and, beyond mainland France and Europe.

## Supporting information

S1 Fig Phylogenetic analysis of DENV1 (A), DENV2 (B), and DENV3 (C) sequences from Reunion Island from 2012 to 2022. Maximum likelihood (ML) trees were constructed to investigate the phylogenetic relationships among these sequences using all sequences from the GenBank database with lengths >5,000 nucleotides. The sequences from Reunion Island obtained in this study are represented by red dots. Branches with a bootstrap value <95% are colored in red.
(DOCX)

S1 Table Primer schemes used in this study. (DOCX)

S2 Table Details of the newly generated DENV sequences. (XLSX)
