# Supplementary figures and images for: Dynamics of emergence and genetic diversity of dengue virus in Reunion Island from 2012 to 2022
Source: PLoS Negl Trop Dis. 2024 May 20;18(5):e0012184. doi: 10.1371/journal.pntd.0012184 (PMC11142707; doi:10.1371/journal.pntd.0012184)

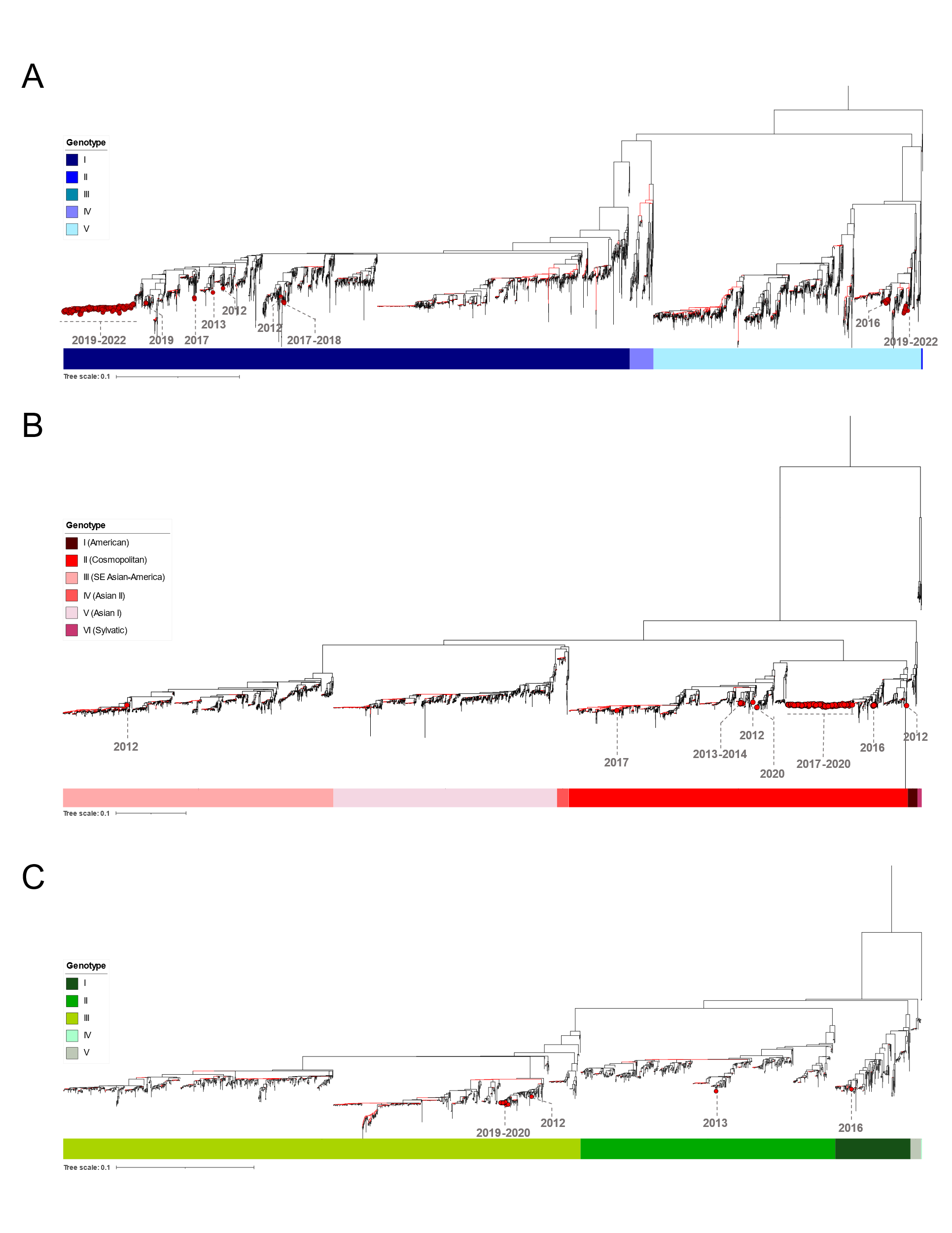


**Supplementary Figure 1**

Supplement: S1 Fig — Maximum likelihood (ML) trees were constructed to investigate the phylogenetic relationships among these sequences using all sequences from the GenBank database with lengths >5,000 nucleotides. The sequences from Reunion Island obtained in this study are represented by red dots. Branches with a bootstrap value <95% are colored in red. (DOCX) [file pntd.0012184.s001.docx]
