# Supplementary material for: Dynamics of emergence and genetic diversity of dengue virus in Reunion Island from 2012 to 2022
Source: PLoS Negl Trop Dis. 2024 May 20;18(5):e0012184. doi: 10.1371/journal.pntd.0012184 (PMC11142707; doi:10.1371/journal.pntd.0012184)
Supplement: S1 Table — (DOCX) [file pntd.0012184.s002.docx]

**Table S1**

List of primers used in this study:

**Dengue Virus 1:**

| **Name** | **Sequence** | **Pool** |
| --- | --- | --- |
| D1V5-1200_1_LEFT | AGCAGATCTCTGATGAACAACCA | 1 |
| D1V5-1200_1_RIGHT | ACACACTTAAACTTGGCACACG | 1 |
| D1V5-1200_2_LEFT | AGATGTCCAACACAAGGAGAAGC | 2 |
| D1V5-1200_2_RIGHT | CCATGTCAGCAGAATCCCTATTCC | 2 |
| D1V5-1200_3_LEFT | AGACACCGCATGGGACTTTG | 1 |
| D1V5-1200_3_RIGHT | CAAGATCTGCAGCACCATTCATG | 1 |
| D1V5-1200_4_LEFT | TATTTCACACAAACAGCAGGGC | 2 |
| D1V5-1200_4_RIGHT | TGGTTCCATCATCTTGGACCTC | 2 |
| D1V5-1200_5_LEFT | TAGCTGGAGGCATGCTAATAGC | 1 |
| D1V5-1200_5_RIGHT | AAGATCGCAGCTGCTTCACC | 1 |
| D1V5-1200_6_LEFT | GTGAGAGTTCCCAATTACAACATGA | 2 |
| D1V5-1200_6_RIGHT | CTCCTTGTTCGGAGTTGTGCA | 2 |
| D1V5-1200_7_LEFT | AGAGTTTGCAGCAGGAAGAAGA | 1 |
| D1V5-1200_7_RIGHT | CCAGACCTGCTCCTGCTAGATA | 1 |
| D1V5-1200_8_LEFT | ATGGGCCTTGTGTGAATCCATC | 2 |
| D1V5-1200_8_RIGHT | GTCATGGCTATTTGTGTGACCAT | 2 |
| D1V5-1200_9_LEFT | ACAAAACATGGGCCTATCATGGA | 1 |
| D1V5-1200_9_RIGHT | TCCATCCTTTTGAAGGTTCCCATT | 1 |
| D1V5-1200_10_LEFT | AAGACAAATGGAGTCTGAGGGAA | 2 |
| D1V5-1200_10_RIGHT | TGGTCTCTCCCAGCGTCAATAT | 2 |

**Dengue Virus 2 :**

| **Name** | **Sequence** | **Pool** |
| --- | --- | --- |
| D2V4-1250_1_LEFT | TCAATATGCTGAAACGCGAGAGA | 1 |
| D2V4-1250_1_RIGHT | TCTTCCCCTGAGTGAGGTGTTA | 1 |
| D2V4-1250_2_LEFT | ACAGAGGATGGGGAAATGGATG | 2 |
| D2V4-1250_2_RIGHT | CCACTGCCACATTTCAGTTCTT | 2 |
| D2V4-1250_3_LEFT | AGTGGGGTCTCATGGACTATGA | 1 |
| D2V4-1250_3_RIGHT | TCCAGGAACAGTGCCATTCC | 1 |
| D2V4-1250_4_LEFT | GAGGTGAGGATGGATGCTGGTA | 2 |
| D2V4-1250_4_RIGHT | TCCTTCTTTGTAAACTCCGGCT | 2 |
| D2V4-1250_5_LEFT | GCATGGTACCTGTGGGAAGT | 1 |
| D2V4-1250_5_RIGHT | TGGCACCCATTTCTGAAATGTC | 1 |
| D2V4-1250_6_LEFT | GGGAAGACTGTTTGGTTTGTTCC | 2 |
| D2V4-1250_6_RIGHT | AAACCCATCTCGTTTGCCATGG | 2 |
| D2V4-1250_7_LEFT | CCACACTGGATAGCAGCTTCAA | 1 |
| D2V4-1250_7_RIGHT | TCGTGTCCTGGTCCTCCTTTT | 1 |
| D2V4-1250_8_LEFT | TCAAAAGAGGAGAAACGGACCA | 2 |
| D2V4-1250_8_RIGHT | CACATGTACCATATGGCTCTGCT | 2 |
| D2V4-1250_9_LEFT | GCACGTGAGGCTGTTGAAGA | 1 |
| D2V4-1250_9_RIGHT | CCGCACCATTGGTCTTCTCTTT | 1 |
| D2V4_1250_10_LEFT | GAAGTCTTACGCCCAAATGTGG | 2 |
| D2V4_1250_10_RIGHT | TCTGTGCCTGGAATGATGCT | 2 |

**Dengue Virus 3 :**

| Name | Sequence | Pool |
| --- | --- | --- |
| D3V2-1200_1_LEFT | AGCAGATCTCTGATGAACAACCA | 1 |
| D3V2-1200_1_RIGHT | CCAAGCTTCCCTTGCCAAACAA | 1 |
| D3V2-1200_2_LEFT | CCTAAGGAAGCTATGCATTGAGGG | 2 |
| D3V2-1200_2_RIGHT | TTTTTGAATTCAACCCTATCCAGGTTA | 2 |
| D3V2-1200_3_LEFT | CATGGCCATCTTGGGAGACA | 1 |
| D3V2-1200_3_RIGHT | CCGTCTTCTCCCATGTATCGCA | 1 |
| D3V2-1200_4_LEFT | TTCAACTATTGTGAAGGAACAACAGT | 2 |
| D3V2-1200_4_RIGHT | AGCACTGTTAGGATGTTCTCAGT | 2 |
| D3V2-1200_5_LEFT | ATAACTGGCACGTCAGCAGAC | 1 |
| D3V2-1200_5_RIGHT | CCTCTCCCATTCCTACACGAGT | 1 |
| D3V2-1200_6_LEFT | TGTTGATCTAATGTGCCACGCA | 2 |
| D3V2-1200_6_RIGHT | TTCCTCCACTGCATGCCTGTA | 2 |
| D3V2-1200_7_LEFT | TGTGACAGAAATAGGAAGAGTGCC | 1 |
| D3V2-1200_7_RIGHT | CCTGTTCCAACTGATTTCATGATAGA | 1 |
| D3V2-1200_8_LEFT | AGGACCAATAACAACACTCTGGG | 2 |
| D3V2-1200_8_RIGHT | GTCATTGCCATCTGTGTCACC | 2 |
| D3V2-1200_9_LEFT | AACCAGAAACACCCAACATGGA | 1 |
| D3V2-1200_9_RIGHT | TTTCACCACGCAATCATCCCC | 1 |
| D3V2-1200_10_LEFT | TCTAGGAAAGACCAAAGAGGCAG | 2 |
| D3V2-1200_10_RIGHT | TCCTCTAACCTCTAGTCCTTTGCA | 2 |
